# A Comparative Evaluation of Three Diagnostic Assays for the Detection of Human Monkeypox

**DOI:** 10.3390/v16081286

**Published:** 2024-08-12

**Authors:** Jing Qu, Xiaomin Zhang, Kun Liu, You Li, Ting Wang, Zhonggang Fang, Cheng Chen, Xiao Tan, Ying Lin, Qing Xu, Yan Yang, Wanqing Wang, Manyu Huang, Shiliang Guo, Ziqiu Chen, Wei Rao, Xiaolu Shi, Bo Peng

**Affiliations:** 1 Microbiology Laboratory, Shenzhen Center for Disease Control and Prevention, Shenzhen 518055, China; qujing861105@163.com (J.Q.); zxm20240808@163.com (X.Z.); yinglin0810@foxmail.com (Y.L.); sevenxu1022@163.com (Q.X.); 15875662596@163.com (Y.Y.); 18086614382@163.com (W.W.); 15977146984@163.com (M.H.); g689235@163.com (S.G.); 13428716465@163.com (Z.C.); 2 Research & Development Department, Shenzhen New Industries Biomedical Engineering Co., Ltd., (Snibe), Shenzhen 518122, China; kun.liu@snibe.cn (K.L.); you01.li@snibe.cn (Y.L.); ting.wang@snibe.cn (T.W.); zhonggang.fang@snibe.cn (Z.F.); cheng02.chen@snibe.cn (C.C.); 3 Shenzhen Biorain Biotechnology Co., Ltd., Shenzhen 518122, China; tanxiaozhiye@gmail.com; 4 National Institute for Viral Disease Control and Prevention, Chinese Center for Disease Control and Prevention, Beijing 102206, China

**Keywords:** monkeypox virus, diagnostics, chemiluminescence immunoassay, droplet digital PCR, real-time qPCR

## Abstract

Accurate and early diagnosis of monkeypox virus (MPXV) is crucial for controlling epidemics and treating affected individuals promptly. This study aimed to assess the analytical and clinical performance of the Molecision^TM^ Monkeypox Virus qPCR Assay, Biorain Monkeypox Virus ddPCR Assay, and MAGLUMI^®^ Monkeypox Virus Ag (chemiluminescence immunoassay, CLIA) Assay. Additionally, it aimed to compare the clinical application of antigen and nucleic acid assays to offer insights into using commercial monkeypox assay kits. Specimens from 117 clinical patients, serial diluted virus cell culture supernatant, and artificially created positive samples were tested to evaluate the performance of these assay kits for MPXV diagnostics. The Biorain Monkeypox Virus ddPCR Assay had a limit of detection (LoD) of 3.89 CCID_50_/mL, while the Molecision^TM^ Monkeypox Virus qPCR Assay had an LoD of 15.55 CCID_50_/mL. The MAGLUMI^®^ Monkeypox Virus Ag (CLIA) Assay had an LoD of 0.500 pg/mL. The accuracy of the Molecision^TM^ Monkeypox Virus qPCR Assay was comparable to the Biorain Monkeypox Virus ddPCR Assay, and the MAGLUMI^®^ Monkeypox Virus Ag (CLIA) Assay demonstrated high sensitivity. The specificity of all three MPXV diagnostic assays for clinical specimens with potential cross-reacting substances was 100%. In conclusion, this study provides valuable insights into the clinical application of monkeypox assays, supporting efforts to mitigate and control the spread of monkeypox.

## 1. Introduction

Monkeypox virus (MPXV) is an *Orthopoxvirus* which causes smallpox-like disease [1]. As of 25 June 2024, over 97,000 cases have been confirmed in the 2022 outbreak, and so far, 207 deaths have been reported globally [2]. Monkeypox (mpox) cases have occurred in more than 110 countries, most of which were reported in non-endemic countries including China [3]. In the recent outbreak, it was reported to be relatively less lethal (0.46%) than the endemic MPXV strains [4]. Global infection cases reached their highest value in August 2022 at the beginning of the outbreak [1]. Currently, two different clades have been reported. Clade I is mainly in Central Africa, especially in the Democratic Republic of the Congo (DRC), and is associated with severe clinical symptoms and substantial mortality (4–11%), whereas clade II was largely confined to West Africa until the 2022 global epidemic and causes less severe illness and lower mortality of <4% [5]. At present, the majority of genetic sequences are associated with clade IIb [6], which caused the ongoing global outbreak of monkeypox spanning from 2022 to 2024 [7]. It is phylogenetically distinct from previously endemic MPXV strains, indicating potential differences in its virological properties [4]. Therefore, identifying nucleotide conservation for future primer design is crucial for nucleic acid assays [8].

MPXV has a double-stranded DNA genome [1]. The disease begins with a febrile prodrome [9]. Skin rashes typically emerge 1–3 days after the onset of fever [10]. Clinical outcomes are usually worse in patients in immunocompromised states, such as bronchopneumonia, encephalitis, and visual loss due to corneal infection [11]. Many of these signs and symptoms are common in various viral and non-viral diseases [10]. Given the atypical clinical presentation, it is important to consider monkeypox in the differential diagnosis [1]. The rash associated with monkeypox may be erroneously identified as chickenpox, shingles, syphilis, herpes, measles, enterovirus, bacterial skin infections (e.g., group A streptococcus [12]), other sexually transmitted infections, or allergies linked to medications [10]. MPXV has shown increased adaptation to humans, increasing the effectiveness of transmission [13]. Although most individuals infected with clade II MPXV recover without treatment since the symptoms of monkeypox infection are typically minor, it is important to note that more than half of cases might been transmitted to others before symptoms appear [14]. The current strategy for post-exposure prophylaxis or pre-exposure prophylaxis for individuals at high risk involves Modified Vaccinia Ankara (MVA)-based vaccination, which has been authorized for emergency use by the Food and Drug Administration (FDA) in the US [15] and the Medicines and Healthcare products Regulatory Agency (MHRA) in the UK [16]. Infections with MPXV do not yet have a specific therapy; however, antiviral drugs that have been licensed to treat smallpox can also be utilized for treating monkeypox [17]. 

In addition to vaccination, rapid diagnosis and infection control measures remain key interventions to reduce ongoing transmission. Verifying MPXV relies on the available laboratory tests in order to control the spread of infection for viral containment [18]. Because of these public health concerns, accurate case identification requires more efficient routine diagnostic testing [19]. The gold standard test to establish the diagnosis is polymerase chain reaction (PCR), such as the real-time quantitative polymerase chain reaction (RT-PCR) system [17]. Nevertheless, the accuracy of the results may be influenced by constraints of sensitivity. The advent of digital droplet PCR (ddPCR), as a third-generation PCR technology, represents a recent innovation for microbiology detection [20]. Although initially applied in poxvirus research [21], this methodology has more recently been adopted for clinical diagnosis of MPXV [22]. PCR requires careful sample handling, sophisticated instrumentation, and time-consuming procedures [23]. Commercial monkeypox detection methods are continually evolving both domestically and internationally, such as PCR assays, serological assays, and viral culture techniques [23]. A multiplexable, magnetic-bead-based Luminex system was developed for the immunoassay [23]. However, commercial immunological methods are insufficient to assist in the diagnosis of MPXV infections through the detection of viral antigens. Low- and middle-income countries need a cheap and rapid diagnostic solution [24], so there is a need for highly sensitive immunological assays, such as a chemiluminescence immunoassay or colloidal gold immunochromatography, that can offer immediate results to clinicians, aiding in their decision-making [17].

The Molecision^TM^ Monkeypox Virus qPCR Assay, MAGLUMI^®^ (Snibe Diagnostics, Shenzhen, China) Monkeypox Virus Ag (chemiluminescence immunoassay, CLIA) Assay, and Biorain Monkeypox Virus ddPCR Assay were developed as MPXV diagnostics. The automated immunoassay detecting viral antigens can rapidly diagnose an active case [25]. And it can be performed with minimal manual operation because of the intelligent MAGLUMI X series analyzers. As for a PCR-based system, conserved genes play crucial roles in key functions such as replication, transcription, and virion assembly. These genes are present and conserved even in new variants or emerging viruses [26]. The Molecision^TM^ Monkeypox Virus qPCR Assay and Biorain Monkeypox Virus ddPCR Assay both target the conserved *F3L* gene of MPXV. In this study, the above commercial assay kits were evaluated for qualifying and quantifying MPXV using human biological specimens, such as vesicular fluid. The analytical and clinical performances were evaluated. Analytical performance included the limit of detection (LoD). Clinical performance included diagnostic specificity and diagnostic sensitivity. Antigen assays and nucleic acid assays were compared and the methodological differences were analyzed.

## 2. Materials and Methods

### 2.1. Sample Origin and Preparation

The evaluation panel we established for precision performance characteristics used synthetic DNA or antigens spiked into MPXV-negative remnant clinical specimens (serum, plasma, and lesion exudate). 

The evaluation panel we established for LoD used DNA from cultured viruses. Virus titers were determined through a CCID_50_ assay on VeroE6 cells obtained from Dr. Liu Yang at Shenzhen Bay Laboratory, Shenzhen, China. Virus stocks were stored in aliquots at −80 degrees until required. The laboratory standard materials we used were purchased from OkayBio (MPXV A35R antigen, catalog number: C1620, Nanjing, China) and Sino Biological (Monkeypox Virus Protein A29, catalog number: 40891-V08E, Beijing, China).

The 117 lesion exudate samples used for this clinical performance study were prepared from the remaining monkeypox virus (hMPXV-1/Shenzhen/27NF/2023/B.1.3) clinical samples that were obtained from patients who were positive for MPXV (determined by RT-PCR). 

### 2.2. Real-Time qPCR Assay

The Molecision^TM^ Monkeypox Virus qPCR Assay kit was manufactured by Shenzhen New Industries Biomedical Engineering Co., Ltd. (Snibe) (Shenzhen, China). 

First, we prepared the reagents according to their instructions for use. This involved adding the reconstitution solution to the internal control and positive control vials and allowing them to rehydrate. Next, we added the extracted DNA from the processed samples to the prepared reagents. We dispensed the samples and controls into the PCR tubes or plate and placed them into the sample tank of the ABI-7500 instrument. Finally, we processed the samples using the following cycling protocol: an initial denaturation step at 95 °C for 5 min, followed by 45 cycles of denaturation at 95 °C for 15 s and annealing/extension at 60 °C for 30 s.

### 2.3. Digital PCR Assay

The Biorain Monkeypox Virus ddPCR Assay kit was manufactured by Shenzhen Biorain Biotechnology Co., Ltd. (Shenzhen, China).

The digital procedure was performed following the manufacturer’s instructions. The FAM probe assay was used for the monkeypox target gene. The CY5 probe assay was used as an internal amplification control. Samples were processed in the DropXpertS6 using the following cycling protocol: 95 °C for 5 min, 95 °C for 15 s for denaturation, and 58 °C for 45 s for annealing/extension for 40 cycles. 

### 2.4. Chemiluminescence Immunoassay

The MAGLUMI^®^ Monkeypox Virus Ag (CLIA) Assay kit was manufactured by Shenzhen New Industries Biomedical Engineering Co., Ltd. (Snibe) (Shenzhen, China). 

According to the manufacturer’s instructions, the experiments were conducted following these steps: First, we prepared the reagents. Next, we calibrated the assay and performed quality control. Finally, we conducted sample testing for 15 min using the MAGLUMI X3 Fully-auto chemiluminescence immunoassay analyzer which required a sample volume of 100 µL for each single determination. A result greater than or equal to 8.0 pg/mL was considered positive.

### 2.5. Analytical and Clinical Performance Study

A positive sample from cultured monkeypox virus was gradient diluted and tested in 20 replicates to estimate the LoD [27]. Precision was determined using the assay, running samples and controls through a protocol (EP05-A3) from the CLSI (Clinical and Laboratory Standards Institute): duplicates in two independent runs per day for 5 days at three different sites using three lots of reagent kits. A total of 117 specimens from clinical patients were tested to estimate the clinical performance of the three different assay kits. The performance characteristics were reported by using the relevant formula together with a 2-sided 95% confidence interval [28]. 

### 2.6. Statistical Analysis

Analytical performance parameters (LoD and precision) and clinical performance parameters (positive percent agreement and negative percent agreement) were analyzed using a diagnostic test evaluation calculator. Comparisons between the results of each assay were performed using the Graphpad Prism software Version 8.3.0. Simple linear regression was used for evaluating the correlation of MPXV DNA measures from the Molecision^TM^ Monkeypox Virus qPCR Assay and Biorain Monkeypox Virus ddPCR Assay [29]. 

## 3. Results

### 3.1. Analytical Performance Evaluation

We assessed the analytical performance of the MAGLUMI^®^ Monkeypox Virus Ag (CLIA) Assay, Molecision^TM^ Monkeypox Virus qPCR Assay, and Biorain Monkeypox Virus ddPCR Assay. In addition to using artificially created positive samples (synthetic DNA or antigens spiked into MPXV-negative remnant clinical specimens), the LoD was determined for β-Propiolactone inactivated (1:1000) cultured virus representing clade IIb 1.3 of an isolate from a Chinese patient. By establishing the LoD in terms of the CCID_50_ study, the sensitivity of these diagnostic assays can be directly related to the infectious viral load. The LoD for the qPCR assay was 200 copies/mL and 15.55 CCID_50_/mL. Meanwhile, the LoD for the ddPCR assay was 80 copies/mL and 3.89 CCID_50_/mL (Supplementary Table S1). Due to the lack of commercially available MPXV antigen standard material, laboratory standard material was used for the LoD study of the CLIA assay. The LoD for the CLIA assay was 0.500 pg/mL.

To assess the precision of the three assays, replicate testing within the same run and in different runs was performed. Generally, all evaluated assays demonstrated good precision. The lowest imprecision was obtained with the qPCR assay, with the within-run precision varying between 1.37% and 1.42%, while the reproducibility ranged from 1.48% to 1.50% (Supplementary Table S2).

### 3.2. Clinical Performance Evaluation

In total, 117 clinical specimens were evaluated by the MAGLUMI^®^ Monkeypox Virus Ag (CLIA) Assay, Molecision^TM^ Monkeypox Virus qPCR Assay, and Biorain Monkeypox Virus ddPCR Assay (Supplementary Figure S1).

MPXV infections among the male population are more frequent than among the female population [30]. According to global surveillance data, the 2022–2023 monkeypox outbreak was driven by transmission among males (73,560 [96.4%] of 76,293 cases) [31]. According to a systematic review, 4152 out of 4222 confirmed cases of monkeypox were male with a mean age of 36 years [32]. In this study, the subject population can represent the characteristics of the target population, that is, demographic characteristics (gender and age). The testing procedure should be verified on a reasonable sample size. Our sample size compares favorably with recent evaluations, as approximately only 10 to 20 MPXV-positive clinical subjects [33] were tested with other MPXV assays in other studies [34]. In this study, 80 were MPXV-positive subjects and 37 were MPXV-negative subjects, and the patient characteristics are demonstrated in Table 1. In this study, 94.87% (111/117) of subjects were male and all subjects were aged between 8 and 51 years, with a median age of 30.74 years. The collected specimens were all from symptomatic patients. 

All three assays demonstrated sensitivity and specificity in detecting lesion exudate specimens, instead of artificial samples, which validated their performance in clinical diagnosis. All three assays were tested for their ability to detect MPXV DNA or antigens using clinical specimens. For a precise analysis of the performance characteristics of the Molecision^TM^ Monkeypox Virus qPCR Assay, we conducted a comparison of the results of the ddPCR assay and qPCR assay, using the results of the ddPCR assay as the standard (Table 2). Overall, the qPCR assay demonstrated a 92.31% overall percent agreement and a 100% negative percent agreement. For all of the above evaluations, negative and positive controls were used and verified.

**Figure 1:**
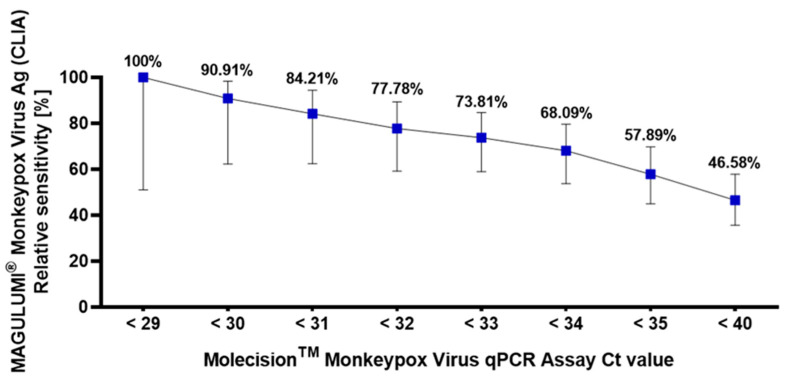
Relative sensitivity of MAGLUMI^®^ Monkeypox Virus Ag (CLIA) Assay in qPCR assay Ct intervals. Each blue square within the illustration signifies the sensitivity of CLIA assay, accompanied by 95% confidence intervals denoted by vertical lines. These values are presented in relation to specific qPCR Ct intervals, including <29, <30, <31, <32, <32, <33, <34, <35, and <40.

Due to methodological differences between the nucleic acid test and the immunological test, we stratified qPCR-positive specimens based on their Ct values (Figure 1) to further assess sensitivity. The MAGLUMI^®^ Monkeypox Virus Ag (CLIA) Assay showed high sensitivity in samples with a cycle threshold value < 30, which was 90.91%. When the cycle threshold value was <29, it showed better sensitivity (100%). 

**Figure 2:**
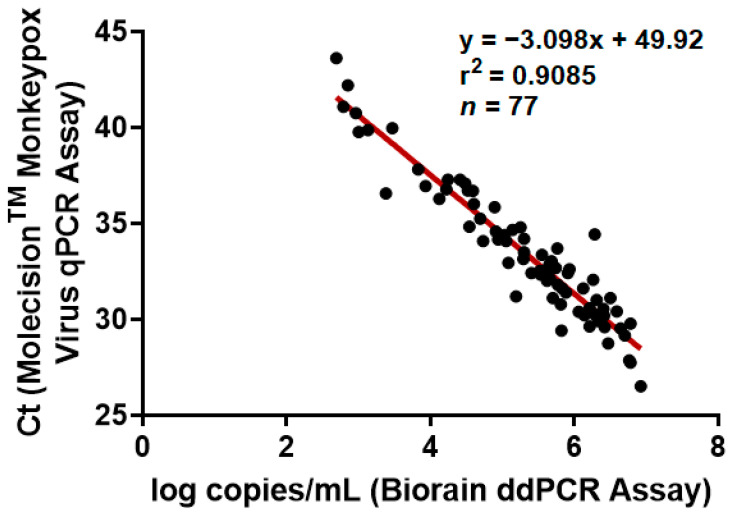
The correlation of MPXV DNA measures from the Molecision^TM^ Monkeypox Virus qPCR Assay/Biorain Monkeypox Virus ddPCR Assay. Scatter plots with a regression line (red line) for the Molecision^TM^ Monkeypox Virus qPCR Assay and Biorain Monkeypox Virus ddPCR Assay. Each black dot symbolizes a specimen both with results from both qPCR assay and ddPCR assay. The results of the ddPCR assay were log-transformed as log_10_ (number of copies/mL) for the purpose of simple linear regression.

The concordance between the qPCR assay and ddPCR assay was 92.31%, with a satisfactory agreement in MPXV detection (Kappa value: 0.8291; 95% CI 0.7219–0.9364; Table 3). A comparative scatter plot of monkeypox viral loads via the Molecision^TM^ Monkeypox Virus qPCR Assay (Ct value) and Biorain Monkeypox Virus ddPCR Assay (log copies/mL) is shown in Figure 2. MPXV levels measured by the Biorain Monkeypox Virus ddPCR Assay were strongly correlated with the Ct values detected by the Molecision^TM^ Monkeypox Virus qPCR Assay (*r* = 0.9085, *p* < 0.0001).

Nonetheless, in this study, all three assays showcased 100% specificity for the 37 specimens, even in the presence of potential cross-reacting substances, such as other pathogens that cause rash symptoms (Table 4). Due to the immunological cross-reactivity of MPXV, many tests relying on antigens lack the necessary specificity [35]. It is noteworthy that the MAGLUMI^®^ Monkeypox Virus Ag (CLIA) Assay showed 100% specificity in accurately diagnosing monkeypox by preventing the generation of false positive results. PCR is currently considered the “gold standard” for laboratory diagnostics today, but CLIA has the potential to serve as a valuable complement tool, given its ease of use and high specificity.

The discrepancy in results (Supplementary Table S3) could be attributed to the higher sensitivity of the ddPCR assay, considering that these specimens (No. 1–No. 8) were at low viral load levels. It is worth noting that half of the discrepancy results (No. 1–No. 4), which were tested by the qPCR assay, marginally exceeded the cut-off Ct values of 40.

### 3.3. Cost-Effectiveness and Time-Effectiveness for Routine Diagnosis of MPXV

The clinical applicability of different platforms for routine diagnosis of MPXV depends on their cost-effectiveness and time-effectiveness. Therefore, all three platforms were assessed in terms of the relative final price per sample (consumables, labor fee, and indirect costs), hands-on time, and turnaround time to conduct the analysis for different numbers of samples (Table 5). Irrespective of performance characteristics, the MAGLUMI^®^ Monkeypox Virus Ag (CLIA) Assay was the most cost-effective and time-effective. Taking sensitivity into account, the Molecision^TM^ Monkeypox Virus qPCR Assay was more suitable for routine diagnosis than the Biorain Monkeypox Virus ddPCR Assay, because the price of the ddPCR assay is around four times higher than that of the qPCR assay and the total turnaround time of the ddPCR assay is two to four times longer than that of the qPCR assay.

## 4. Discussion

Due to the emergence of the new monkeypox pandemic and the fact that clinical manifestations caused by different *Orthopoxviruses* are similar, identifying monkeypox based on symptoms alone is challenging [36]. Therefore, early detection can help to identify infected people and alert them to take timely isolation and treatment measures, thereby reducing the spread of the virus and mitigating the impact of the outbreak [35]. We evaluated three MPXV diagnostic assays characterized by sensitivity, specificity, accuracy, effectiveness, and accessibility. The initiative is intended to enhance the precise identification of the infection, thereby aiding in the containment of widespread diseases within the public health sector and promoting health protection measures. 

Generally, all evaluated assays demonstrated good analytical performance. The CCID_50_ study provides a direct link between the assays’ ability to detect MPXV and the infectious potential of the specimens, which is crucial for clinical decision-making. We have demonstrated the LoD of the Molecision^TM^ Monkeypox Virus qPCR Assay to be as low as 200 copies/mL and 15.55 CCID_50_/mL. Our analytical sensitivity is comparable to some recent evaluations of other commercial PCR assays, which also use the *F3L* gene as their target [37]. The LoD of the Biorain Monkeypox Virus ddPCR Assay obtained with the cultured virus for the *F3L* gene was 80 copies/mL and 3.89 CCID_50_/mL. The droplet digital PCR assay exhibited a lower LoD and may provide an opportunity to improve the diagnostic sensitivity currently seen using the qPCR assay. 

The adequate sample size of this study enables the scientific determination of the sensitivity and specificity of the Sinbe MPXV diagnostic platform for clinical specimens. This study evaluated 117 specimens from clinical patients using three different assay kits. The accuracy of the qPCR assay was found to be comparable to that of the ddPCR assay. Furthermore, all three MPXV diagnostic tests demonstrated 100% specificity for clinical specimens with potential cross-reacting substances. This is attributed to the distinct target sequences of the qPCR assay and ddPCR assay, which focus on the *F3L* gene in MPXV.

The CLIA assay utilized in this study was developed based on the double-MPXV monoclonal antibody, which specifically targets the A29 and A35R epitopes. Roumillat et al. identified a monoclonal Ab (mAb 69-126-3-7) that exhibited binding capabilities toward MPXV, albeit without the ability to neutralize it [38]. The A35R protein, a crucial component of the extracellular enveloped virus, plays an important role in MPXV transmission [39]. Therefore, targeting the MPXV A29 and A35R proteins is essential for ensuring accurate diagnostic outcomes. The limitations of this study include the challenges posed by the conservation of antigens within orthopoxvirus genomes [40], the limited prevalence of other orthopoxviruses, and the scarcity of orthopoxvirus-positive specimens. As a result, the CLIA assay may not be able to distinguish effectively between MPXV and other specific species of orthopoxviruses. 

The rising incidence of clade I MPXV infectious in Central Africa [6], particularly in isolated forest regions, is believed to be attributed to zoonotic transmission events, leading to subsequent human-to-human spread within households [41]. The lack of available clinical samples from clade I MPXV cases in the DRC hinders our ability to confirm these occurrences. The validation of clade I MPXV infections will be pursued through the implementation of these three assays in future studies.

The response to the ongoing monkeypox epidemic necessitates the availability of diagnostics that enable rapid detection of MPXV from clinical specimens [19]. Fully automated sample-to-result systems are required. The chemiluminescence immunoassay is not as sensitive as molecular diagnostic assays, but minimal sample manipulation reduces the risks of exposure and contamination of laboratory staff. Given its high diagnostic specificity, convenience, and effectiveness, rapid detection systems using the automated CLIA analyzer MAGLUMI X3 are more compatible with small- and medium-sized hospitals and labs in developing regions where resources for viral nucleic acid assays are limited. 

In addition, novel robust techniques with high sensitivity and specificity rates are required, not only for MPXV diagnostic purposes, but also for monitoring viral load. The Biorain Monkeypox Virus ddPCR Assay was developed for patient follow-up and frequent viral load monitoring. Compared to the quantitative ddPCR assay, the Molecision^TM^ Monkeypox Virus qPCR Assay is characterized by its ease of operation and user-friendly control, particularly with the utilization of lyophilized bead regents for the detection of MPXV DNA. This innovation offers comparable or enhanced results compared to conventional wet reagents. A solid-state reagent possessing robust characteristics addresses the challenges associated with PCR reagent delivery and storage, reducing the need for cold chain transportation [42]. The incremental advancements in the existing technology, coupled with thorough validation, can improve the performance and user-friendliness of the MPXV test in the diagnostic laboratory. 

Overall, the three MPXV diagnostic assays we evaluated, which integrate both antigen and nucleic acid assays, exhibit a versatile range of applications. This innovative platform can be switched adaptably to meet the specific requirements of diverse clinical scenarios. Its flexibility allows for targeted and efficient utilization, enhancing its suitability across a spectrum of diagnostic needs in various medical settings.

## Figures and Tables

**Table 1:** Subject descriptors.

Descriptive Statistics		All (N = 117)	MPXV-Positive Patients (N = 80)	MPXV-Negative Patients (N = 37)
Age	Mean (SD)	30.74 (7.3)	31.99 (7.2)	28.05 (7.1)
	Min–max	8–51	20–51	8–44
Gender	F	6	0	6
	M	111	80	31

**Table 2:** Performance characteristics of Molecision^TM^ Monkeypox Virus qPCR Assay.

Performance Characteristics	Molecision^TM^ Monkeypox Virus qPCR Assay
Overall Percent Agreement (95% CI)	92.31% (86.02–95.90%)
Positive Percent Agreement (95% CI)	89.02% (80.44–94.12%)
Negative Percent Agreement (95% CI)	100% (90.11–100.00%)
NPV (95% CI)	79.55% (65.50–88.85%)

**Table 5:** Comparison of cost-effectiveness and time-effectiveness among three assays for diagnosis of clinical samples.

Number of Samples	Items	MAGLUMI^®^ Monkeypox Virus Ag (CLIA) Assay	Molecision^TM^ Monkeypox Virus qPCR Assay	Biorain Monkeypox Virus ddPCR Assay
1	Relative final price per sample (%)	100	200~250	1000~1200
	Hands-on time (h)	0.05	0.20	0.50
	Total turnaround time	0.22	1.70	3.60
10	Relative final price per sample (%)	100	200~250	1000~1100
	Hands-on time (h)	0.10	0.30	0.75
	Total turnaround time	0.50	1.80	3.85
20	Relative final price per sample (%)	100	200~250	1000~1100
	Hands-on time (h)	0.12	0.50	1.5
	Total turnaround time	0.58	2.00	7.5

**Table 3:** Concordance of MPXV DNA results from Molecision^TM^ Monkeypox Virus qPCR Assay and Biorain Monkeypox Virus ddPCR Assay.

Molecision^TM^ Monkeypox Virus qPCR Assay	No. Examined	Biorain Monkeypox Virus ddPCR Assay		Kappa Value (95% CI)
		Positive	Negative	
Positive	73	73	0	0.8291
Negative	44	9	35	(0.7219–0.9364)

**Table 4:** Specificity of three MPXV diagnostic assays for specimens with potentially cross-reacting substances.

Clinical Category	Molecision^TM^ Monkeypox Virus qPCR Assay		MAGLUMI^®^ Monkeypox Virus Ag (CLIA) Assay		Biorain Monkeypox Virus ddPCR Assay	
	R ^1^	NR ^2^	R	NR	R	NR
Rubella virus	0	7	0	7	0	7
Herpes simplex virus-1	0	5	0	5	0	5
Herpes simplex virus-2	0	6	0	6	0	6
Varicella virus	0	7	0	7	0	7
Treponema pallidum	0	6	0	6	0	6
Human papillomavirus	0	5	0	5	0	5
Measles virus	0	1	0	1	0	1
Total	0	37	0	37	0	37
Specificity	100%		100%		100%	
95% CI	90.11–100.00%		90.11–100.00%		90.11–100.00%	

^1^ R, reactive. ^2^ NR, non-reactive.

## Data Availability

Data is contained within the article or Supplementary Material. The original contributions presented in the study are included in the article/Supplementary Materials, further inquiries can be directed to the corresponding authors.

## Supplementary Materials

The following supporting information can be downloaded at https://www.mdpi.com/article/10.3390/v16081286/s1, Figure S1: Analytical samples and clinical specimens evaluated by three MPXV diagnostic assays; Table S1: LoD determination of qPCR assay and ddPCR assay; Table S2: Precision for MPXV determinations with three assays; Table S3: Discrepancy results.
