# Supplementary material for: A Comparative Evaluation of Three Diagnostic Assays for the Detection of Human Monkeypox
Source: Viruses. 2024 Aug 12;16(8):1286. doi: 10.3390/v16081286 (PMC11360518; doi:10.3390/v16081286)
Supplement: Supplementary file 1 [file viruses-16-01286-s001.zip › viruses-3112615-supplementary.pdf]

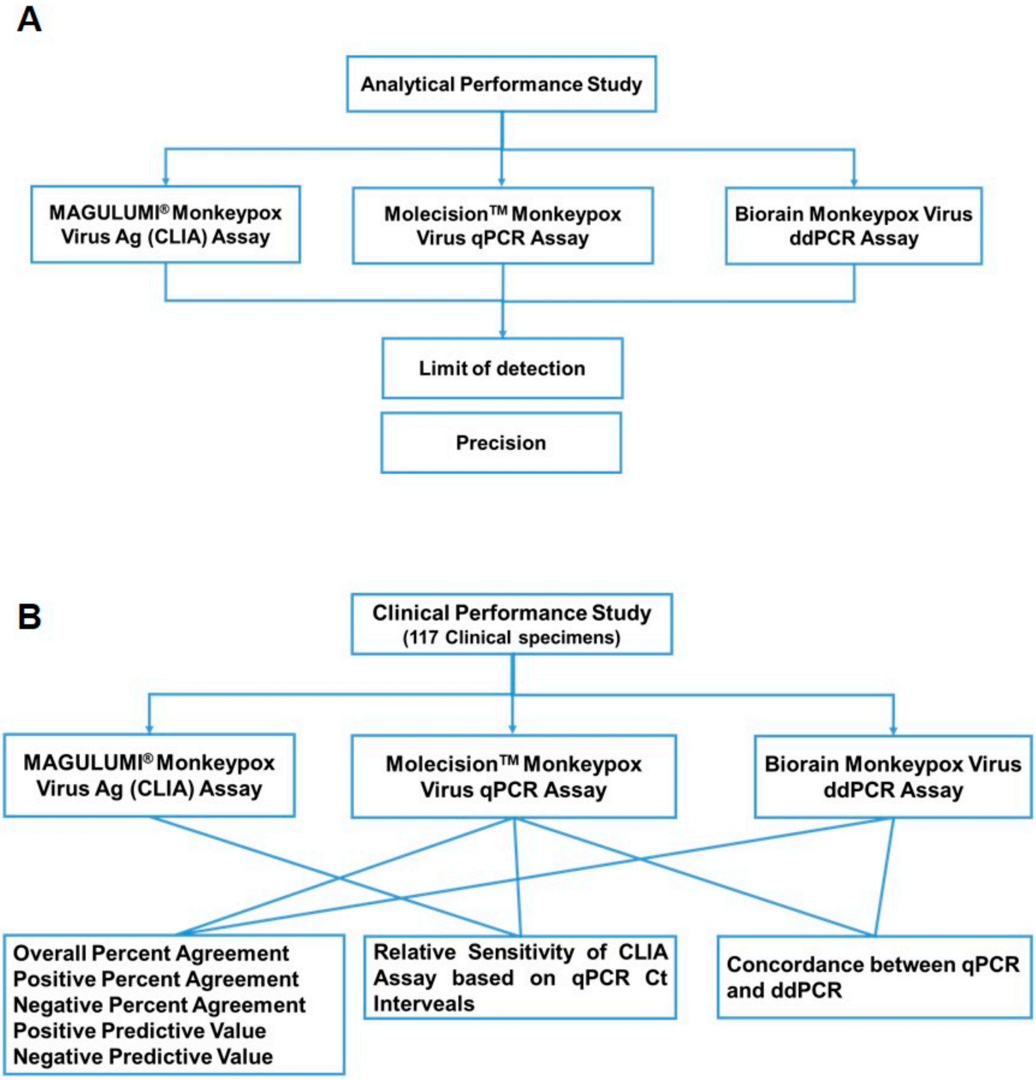

Figure S1 Analytical samples and clinical specimens evaluated by three MPXV Diagnostic Assays

Table S1 LoD determination of qPCR Assay and ddPCR Assay

Molecision™ Monkeypox Virus qPCR Assay		Biorain Monkeypox Virus ddPCR Assay	
Concentration ($TCID_{50}$/mL)	MPXV Hit Rate	Concentration ($TCID_{50}$/mL)	MPXV Hit Rate
62.20	100% (20/20)	15.55	100% (20/20)
31.10	100% (20/20)	7.78	100% (20/20)
15.55	95% (19/20)	3.89	95% (19/20)
7.78	90% (18/20)	1.94	35% (7/20)
3.89	90% (18/20)	0.97	20% (4/20)

Table S2 Precision for MPXV determinations with three assays.

	Sample	Number of valid replicates	Within-run (%CV)	Reproducibility (%CV)
MAGLUMI® Monkeypox Virus Ag (CLIA) Assay	Serum Pool 1	180	3.10	4.65
	Serum Pool 2	180	2.12	3.84
	Serum Pool 3	180	1.40	2.02
	Plasma Pool 1	180	2.52	3.26
	Plasma Pool 2	180	2.42	3.34
	Plasma Pool 3	180	1.52	1.96
	Lesion Exudate Pool 1	180	2.54	4.80
	Lesion Exudate Pool 2	180	2.38	3.42
	Lesion Exudate Pool 3	180	1.20	2.31
MolecisionTM Monkeypox Virus qPCR Assay	Positive Sample	90	1.37	1.48
	Low Positive Sample	90	1.42	1.50
Biorain Monkeypox Virus ddPCR Assay	Positive Sample	30	3.90	5.01
	Low Positive Sample	30	4.20	6.64

Table S3 Discrepancy Results

No.	Sample ID	MolecisionTM Monkeypox Virus qPCR Assay (Ct)	Biorain Monkeypox Virus ddPCR Assay (log copies/mL)
1	KY149	N [1] (42.21)	P [2] (2.61)
2	KY54	N (43.63)	P (2.69)
3	KY341	N (40.76)	P (2.96)
4	KY145	N (41.09)	P (2.79)
5	KY201	N (Undetermined)	P (2.91)
6	KY231	N (Undetermined)	P (2.70)
7	KY246	N (Undetermined)	P (3.06)
8	KY286	N (Undetermined)	P (2.46)

[1] N, negative

[2] P, positive
